# A Fe-C-Ca big cycle in modern carbon-intensive industries: toward emission reduction and resource utilization

**DOI:** 10.1038/srep22323

**Published:** 2016-02-29

**Authors:** Yongqi Sun, Seetharaman Sridhar, Seshadri Seetharaman, Hao Wang, Lili Liu, Xidong Wang, Zuotai Zhang

**Affiliations:** 1 Department of Energy and Resources Engineering, College of Engineering, Peking University, Beijing 100871, P.R. China; 2 WMG, International Digital Laboratory, University of Warwick, Coventry CV4 7AL, UK; 3 Department of Materials Science and Engineering, Royal Institute of Technology, Stockholm, Vallslingan 14, SE-187 52 Täby, Sweden; 4 School of Environmental Science and Engineering, South University of Science and Technology of China, Shenzhen, P.R.China

## Abstract

Herein a big Fe-C-Ca cycle, clarifying the basic element flows and energy flows in modern carbon-intensive industries including the metallurgical industry and the cement industry, was proposed for the first time in the contexts of emission reduction and iron ore degradation nowadays. This big cycle was focused on three industrial elements of Fe, C and Ca and thus it mainly comprised three interdependent loops, i.e., a C-cycle, a Fe-cycle and a Ca-path. As exemplified, we started from the integrated disposal of hot steel slags, a man-made iron resource via char gasification and the employment of hematite, a natural iron resource greatly extended the application area of this idea. Accordingly, based on this concept, the theoretical potentials for energy saving, emission reduction and Fe resource recovery achieved in modern industry are estimated up to 7.66 Mt of standard coal, 63.9 Mt of CO_2_ and 25.2 Mt of pig iron, respectively.

Nowadays global warming has been one of the most significant issues faced by modern society and the globe must limit its future carbon emission to around 1 trillion tonnes to keep global warming within 2 °C over the pre-industrial levels^1^,^2^. China is responsible for ~25% of global carbon emissions, with the total CO_2_ production of 2.49 gigatonnes (Gt) in 2013 according to a recent estimate^3^, a high level approaching the European average. And the carbon emission in China mainly resulted from two parts, i.e., fossil fuel combustion (90%) and cement production (10%) and for the former part, the metallurgical industry contributed to around 12% of total carbon emission^4^,^5^. Recently China has set ambitious target to peak its carbon emission by 2030, which contributes to a major force behind the effort to establish an effective mitigation^2^,^6^. On the way towards low carbon emission, implementing novel technologies to upgrade the traditional carbon-intensive industries, such as the metallurgical industry^7^, accounts for a key strategy.

In 2014, the total output of crude steel in China were ~823 million tonnes (Mt), accounting for world’s half production^8^, and correspondingly, about 123 Mt steel slags were discharged in the metallurgical industry. On one hand, steel slags, tapped at temperatures of 1450–1650 °C^9^,^10^,^11^,^12^, carry enormous high-grade thermal energy of 1.91*10^14^ J, equivalent to 6.52 Mt standard coals. However, most of the high temperature heat is wasted with a low recovery ratio of 2%^13^ because of the fundamental constraints such as low thermal conductivity and high crystallization trend of steel slags^9^,^10^,^14^. To meet these challenges, extensive approaches have been developed, amongst which chemical method offers significant advantages such as production of high value syngas and integration of multiple sectors^11^,^14^. Here an emerging strategy, char gasification, was performed as the first step toward recovering the waste heat from steel slags.

On the other hand, these slags, mainly composed of CaO, Fe_x_O_y_, SiO_2_, Al_2_O_3_ and MgO^9^,^10^,^11^, made up an important material resource for the steel industry and cement industry. In particular, the content of Fe_x_O_y_, mainly in form of FeO, are around 25%^15^,^16^, and thus the iron tapped are up to 23 Mt annually in steel slags, which accounted for an important iron resource. However, this great amount of slags is generally discarded naturally in slag yards, leading to a great wastage of iron resource^10^,^11^,^12^. Meanwhile, the global iron ore has been greatly degraded recently^17^,^18^, which increases the costs of ironmaking and steelmaking and thus necessitates the utilization of low-grade iron resources such as steel slags, hematite and limonite; this, in fact, accounted for an important motivation of the present study.

Furthermore, during the char gasification integrated with steel slag disposal, the transient and final valence states of Fe and C elements are still unclear, especially under a non-equilibrium condition in a flowing gasifying agent. The clarification of this could provide significant clues of Fe and C flows in the metallurgical industry. In addition, the further utilization of the solid wastes, after iron extraction and heat recovery, should also be taken into account, which, in fact, offered the basic information of Ca flows in modern industry. These three elements, Fe, C and Ca, accounted for the fundamental elements in modern carbon-intensive industries. However, the fundamental flows for these elements, especially simultaneously in a big cycle, have not been clearly clarified especially in the context of energy saving and material recycling.

The present study was thus motivated with respects to the two great issues, i.e., emission reduction and resource utilization. To deal with these issues, we began with the heat recovery and material recycling of steel slags, a man-made iron resource, using char gasification reaction (coal char and biomass char) and then a natural iron resource, hematite, was further employed to explore a promising way towards utilization of iron resources. In the end, a big idea of Fe-C-Ca cycle was proposed where the development of modern industry was reconsidered including the metallurgical industry and the cement industry.

## Results

### Route of integrated utilization of steel slags via gasification of coal char

This study started from the treatment of steel slags using coal char gasification and the initial analysis was thus focused on identification of the gasification process and the role of steel slags, especially on the Boudouard reaction expressed as follows:


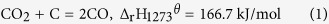


It should be pointed out that the thermodynamic values of the reactions given in this study were calculated using the FactSage software^19^ under the conditions of atmospheric pressure and the temperature of 1273 K.

### Role of steel slags on coal char gasification

**Figure 1:**
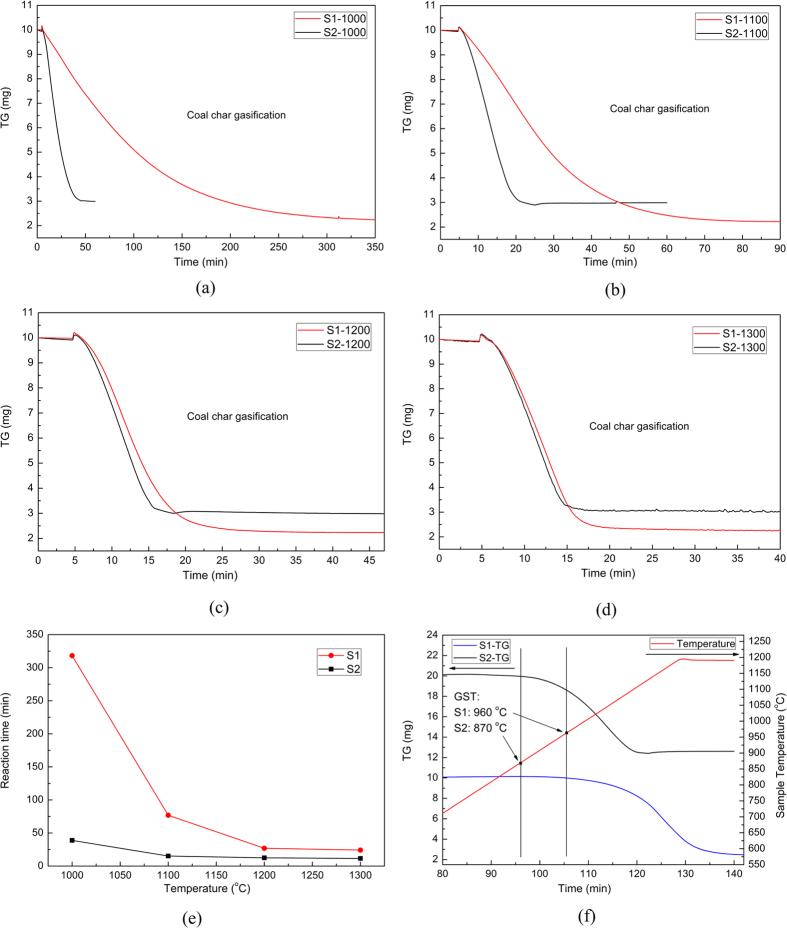
Gasification of coal char with steel slags. (**a**) 1000 °C, (**b**) 1100 °C, (**c**) 1200 °C, (**d**) 1300 °C, (**e**) reaction time, and (**f**) non-isothermal gasification with the heating rate of 10 °C/min. Sample **S1**: raw coal char; Sample **S2**: coal char mixed with steel slags; **GST**: gasification starting temperature.

As the coal char/CO_2_ reaction occurred at high temperatures, the sample mass would continuously vary, which was simultaneously detected using a precision balance. The mass evolutions of these isothermal experiments are displayed in Fig. 1 and supplementary Fig. 1, based on which several characteristics could be clarified. First, supplementary Fig. 1a,b showed that the gasification reaction was greatly enhanced with increasing gasifying temperatures especially for the raw coal char without slags due to a promoting rate at high temperatures. Second, as seen from Fig. 1a–e, the steel slags prominently improved the char gasification manifested by a higher reaction rate and a shorter reaction time, especially at lower temperatures when the intrinsic gasification rate was quite low. This indicated that the steel slags could act as an effective catalyst for char gasification.

To further identify the influence of steel slags, a non-isothermal experiment was conducted at a heating rate of 10 °C/min under pure CO_2_ and the results are illustrated in Fig. 1f. Similar to the isothermal experiments, the gasification time was remarkably shortened by steel slags. Moreover, the temperature when gasification started was pronouncedly lowered by the steel slags, i.e., from 960 °C to 870 °C, which further proved that the steel slags improved the activity of coal char and thus enhanced the reactivity of gasification.

### Characterization of coal char gasification

**Figure 2:**
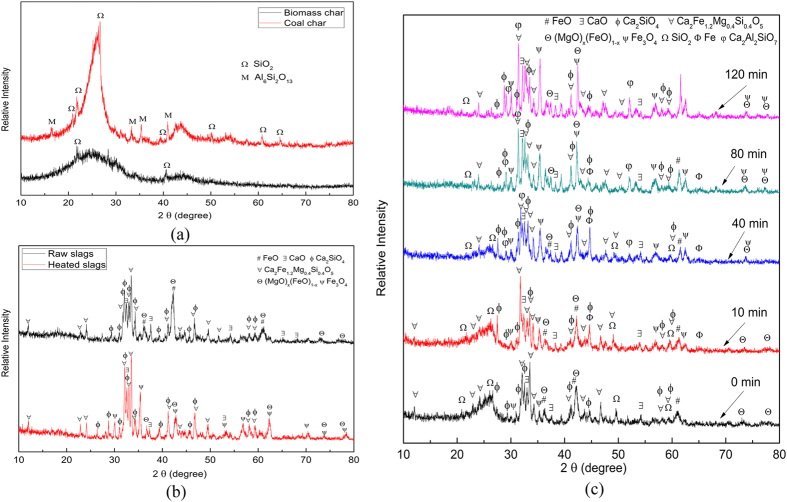
XRD characterizations of the gasification process. (**a**) chars employed, (**b**) raw slags and heated slags in the atmosphere of CO_2_, and (**c**) residual solids.

To further determine the mechanism of char gasification and the impact of steel slags, a series of quenching experiments were performed where the transient state of the process was recorded. The samples obtained this way were analyzed by X-ray powder diffraction (XRD) techniques and the results are detailed in Fig. 2. Firstly, it can be observed that the amorphous envelops of the char in the 2θ range of 20–30 °C^20^,^21^ gradually decayed with increasing reaction time, which revealed that the char was stepwise consumed as the gasification progressed. Secondly, the Fe elements in the slags before gasification were mainly distributed in three mineral phases, i.e., FeO, spinel ((MgO)_x_(FeO)_1−x_) and Ca_2_Fe_1.2_Mg_0.4_Si_0.4_O_5_ and the latter two phases slightly changed during the gasification reactions. Thirdly, the content of Fe_3_O_4_ phase in the solid wastes was remarkably increased, indicating that FeO in the slags was oxidized into Fe_3_O_4_ under the present experimental conditions, as described by means of Eq. (2). This provided an important clue of Fe recovery from the steel slags, i.e., FeO was first oxidized into Fe_3_O_4_ and then extracted via magnetic separation. Actually, in 2012, Matsuura *et al.*^22^ calculated the thermodynamics of H_2_ generation by reaction between FeO in the steel slags and steam in the H_2_O-Ar mixture using the waste heat and meanwhile Sato *et al.*^23^ designed an experimental apparatus to realize it in the lab-scale. This, in fact, realized and exemplified part of the ideas of Fe-C-Ca big cycle in the present study. In particular, as raw steel slags was heated under the agent of CO_2_, the formation of Fe_3_O_4_ and the release of CO gas were directly detected, as evident from Fig. 2b and supplementary Fig. 2; this also indicated the existence of reaction (2).


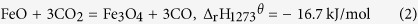


In addition, from Fig. 2c an interesting phenomenon could be observed that Fe phase was transiently formed during the gasification process which could be caused by two factors. Firstly, because of the resistance to gas diffusion, there may not be enough CO_2_ in the gas atmosphere locally and therefore the FeO in the slags was first reduced into Fe. Secondly, although there was CO_2_ in the local position, it was rapidly consumed during the char/CO_2_ reaction, the local CO_2_ content was thus quite low and consequently, the FeO was reduced into Fe by the local fixed carbon. With the reaction proceeding, the fixed carbon in the char run out and the Fe phase was finally oxidized into Fe_3_O_4_ by the CO_2_ agent, as presented in the XRD results.

### Syngas production during coal char gasification

**Figure 3:**
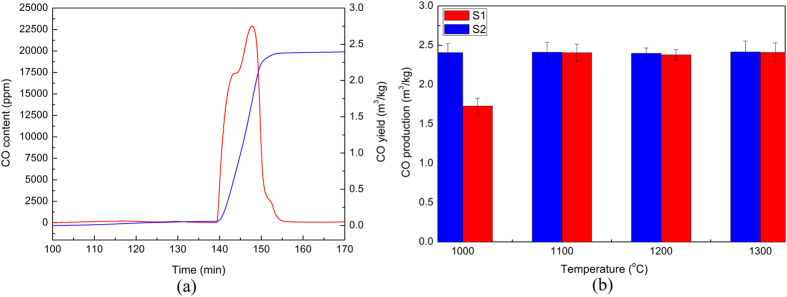
CO productions during the coal char gasification. (**a**) transient CO content and integral CO production at 1200 °C, and (**b**) CO yields with varying temperatures. Sample **S1**: raw coal char; Sample **S2**: coal char mixed with steel slags.

As one of the main objectives of the char gasification reaction was to produce the syngas, thus the CO yield was calculated based on the transient CO content curves, as sketched in Fig. 3a. It should be pointed out herein that the apparent kinetic mechanism of gasification reaction could also be identified based on the transient CO curves, which offered the information of conversion degree versus time in terms of syngas release^24^,^25^,^26^. However, as the mass evolution of the sample versus time was also detected through a thermo-gravimetric (TG) analyzer in the present study, thus the kinetic mechanism were mainly determined based on the TG curves, as discussed later, which, nevertheless, did not weaken the significance of these curves of CO content.

The results of CO productions with varying temperature are presented in Fig. 3b. As can be seen, in case of coal char gasification without steel slags, the CO production at 1000 °C was greatly less than that with slag additions because of the substantial residual char in the solid wastes. This indicated that the presence of the steel slags not only improved the activity of the char gasification but also decreased the residual char and thus increased the CO production at low temperatures. As the temperature was higher than 1100 °C, the CO production did not remarkably change with increasing temperature because the reaction activity was quite high at high temperatures and thus the content of residual char was quite low in the solid wastes after gasification. Another phenomenon was that the CO production with steel slags was slightly more than that without steel slags, which, actually, could stem from the Fe-C reaction expressed by Eq. (2).

### Kinetic mechanisms of coal char gasification

**Figure 4:**
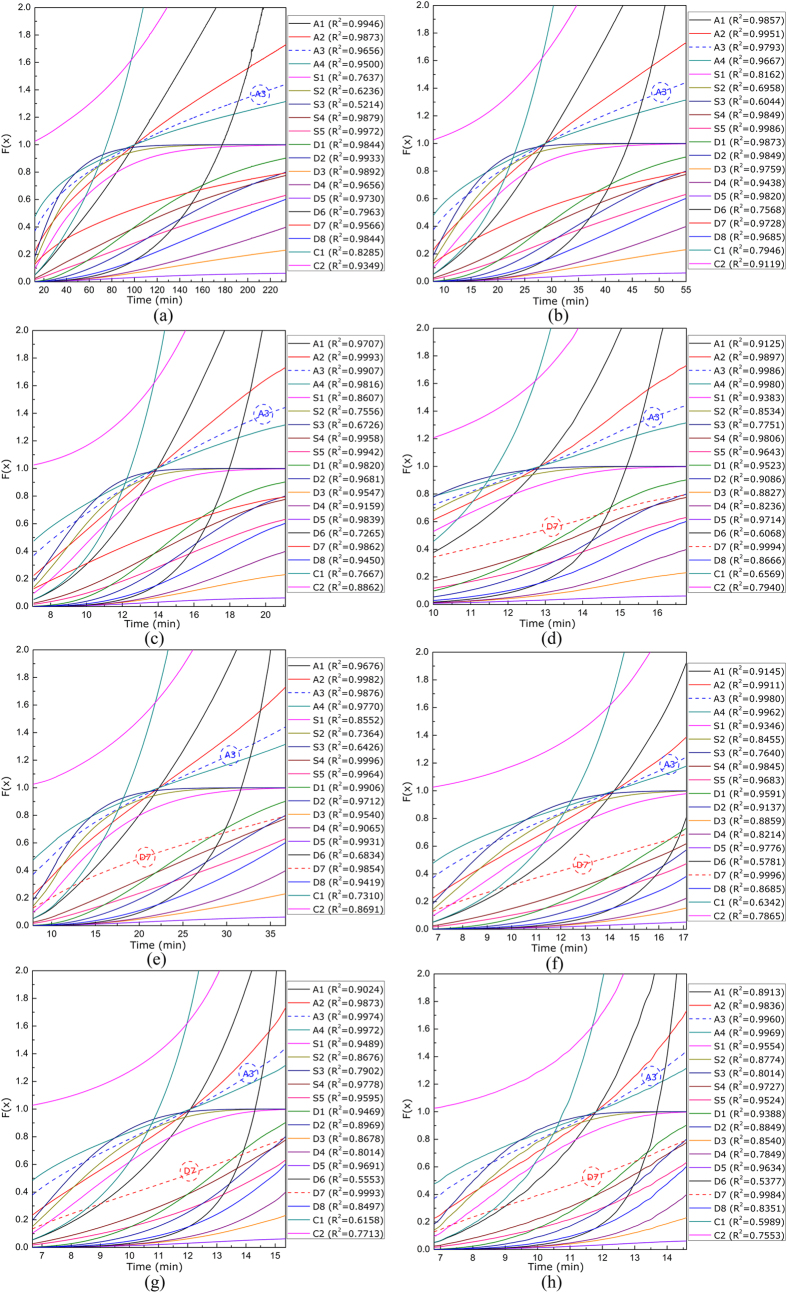
Kinetic mechanism of coal char gasification (**a–d**) without steel slags (**S1**) and (**e–h**) with steel slags (**S2**). (**a**) **S1** at 1000 °C, (**b**) **S1** at 1100 °C, (**c**) **S1** at 1200 °C, (**d**) **S1** at 1300 °C, (**e**) **S2** at 1000 °C, (**f**) **S2** at 1100 °C, (**g**) **S2** at 1200 °C, and (**h**) **S2** at 1300 °C.

To clarify the kinetics of char gasification process, numerous mechanism models including nucleation growth, chemical reaction and mass diffusions^27^,^28^,^29^, were adopted to fit the data of conversion degree versus time, as plotted in Fig. 4. From the viewpoint of linear relationship in the entire temperature range, an A3 model (Avrami-Erofeev) could best interpret the char gasification process both for the raw coal char and the mixture of coal char and steel slags, as described by Eq. (3). This was consistent with the results from previous studies^28^,^29^,^30^,^31^ that Avrami-Erofeev model could be used to interpret the coal gasification process either based on the mass evolution data or based on the transient syngas content data. With the char reaction progressing, coal ash would separate from the fixed carbon and thus the porosity of the sample would vary gradually; and it was therefore scientific to apply an Avrami-Erofeev model to interpret the char reactions.


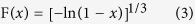


On the other hand, as for the gasification of raw coal char, a D7 model (3-D, Jander), described by Eq. (4), also showed a good linear relationship at 1300 °C, suggesting that the gas diffusion stage could become a dominant step with increasing surficial reaction rate at high temperatures. Especially for the gasification of char/slag mixture, the D7 model offered a good linear relationship at all gasifying temperatures, which unequivocally indicated that the addition of steel slags remarkably improved the surficial reaction rate and thus the mass diffusion step could gradually accounted for a determining step.


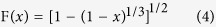


After figuring out the kinetic model of char gasification process, the rate constants could be deduced and consequently, the apparent activation energy for char gasification could then be calculated using Arrhenius equation, as detailed in supplementary Fig. 3 and Table 1. The apparent activation energy greatly decreased from 185.05 kJ/mol to 72.73 kJ/mol, which undoubtedly demonstrated the catalytic effect of steel slags. On the other hand, according to the classical theory of gas-solid reaction^32^,^33^, the activation energy of raw coal char gasification, 185.05 kJ/mol, was in the field of surficial reaction-controlling; while the smaller activation energy with steel slags, 72.73 kJ/mol, revealed that the gasification process could be greatly influenced by the step of mass diffusion. This agreed well with the fact that a D7 model offered a good linear relationship in the presence of steel slags. Since there existed free CaO and FeO in the steel slags and the mineral phases in the slags could be rewritten as M_x_O_y_ · aSiO_2_ · bAl_2_O_3_, the overall catalytic effect of the steel slags could thus be elucidated as follows^34^,^35^:


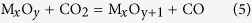



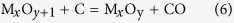


### Route of integrated utilization of steel slags via gasification of biomass char

**Figure 5:**
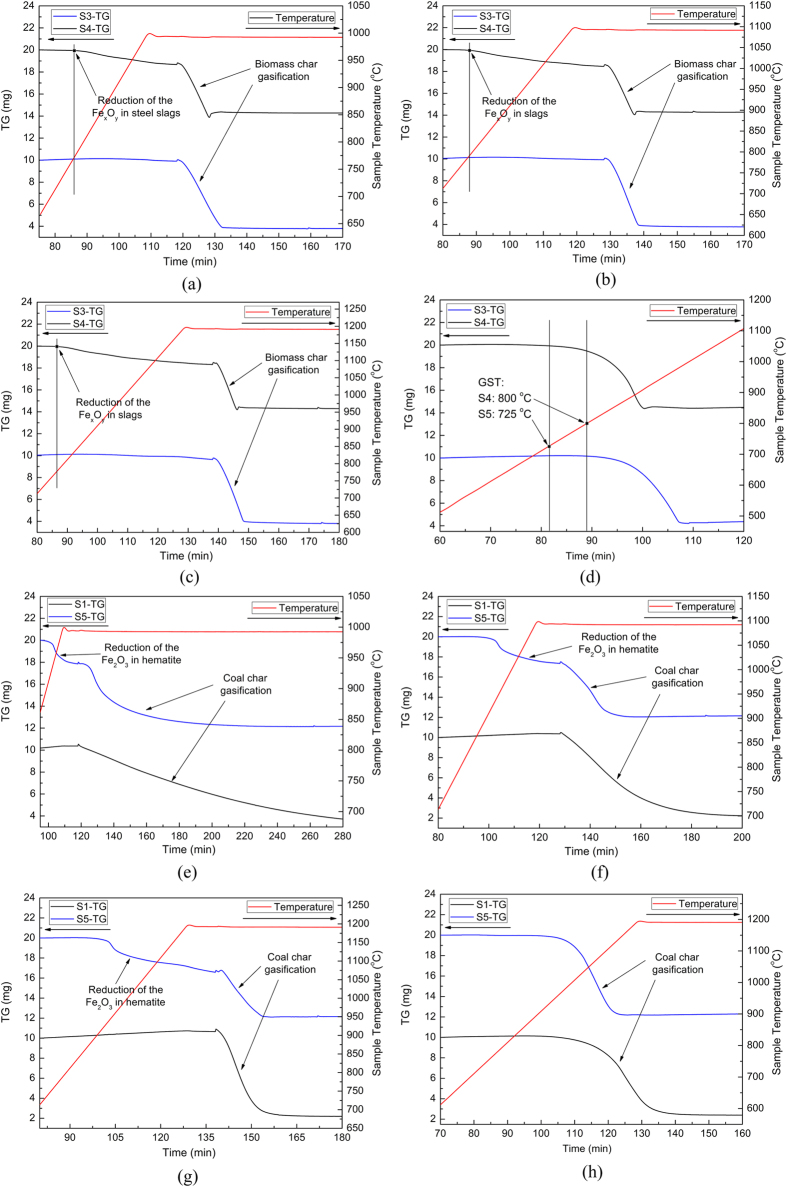
Extended gasification experiments. (**a–d**) gasification of biomass char with steel slags from 1000 to 1300 °C and (**e–h**) gasification of coal char with hematite from 1000 to 1300 °C. Sample **S1**: raw coal char; Sample **S3**: raw biomass char; Sample **S4**: biomass char mixed with steel slags; Sample **S5**: coal char mixed with hematite; **GST**: gasification starting temperature.

In addition to coal char, another kind of char, biomass char, was utilized for disposal of steel slags in this study and the mass evolutions during isothermal gasification are detailed in Fig. 5a–c. It can be observed that the sample mass started to decrease at relatively low temperatures (~770 °C) under Ar gas, indicating that the Fe_x_O_y_ in steel slags was reduced by the carbon in biomass char. This, in fact, accounts for one of the important iron recovery approaches from steel slags, i.e., direct reduction^36^,^37^. This phenomenon also indicated that, from the viewpoint of Fe_x_O_y_ reduction in steel slags, biomass char showed a higher reaction activity than coal char.

To further clarify the role of steel slags on biomass char gasification, a non-isothermal experiment under pure CO_2_ were then conducted with a heating rate of 10 °C/min, as displayed in Fig. 5d. As can be observed, the biomass char gasification was greatly improved by the steel slags, i.e., not only the temperature that the gasification reaction started was lower with slag additions (from 800 °C to 725 °C) but also the reaction time was slightly shortened. This also revealed an obvious catalytic effect of steel slags on biomass char gasification. Additionally, an interesting phenomenon was observed that the gasification temperature of biomass where the reaction started was remarkably lower than that of coal in Fig. 1f, which indicated a higher gasification reactivity of biomass char than coal char due to the different compositions of ashes in the chars^38^. In all, if biomass char gasification was performed for heat recovery from steel slags, the gasifying agent should be accurately controlled as pure CO_2_ based on the present results and the Fe-C-O phase diagram in supplementary Fig. 4.

### Hematite utilization integrated with coal char gasification

Steel slags could be initially taken as a kind of man-made iron resource and to expand the utilization area of the present thought proposed, a natural iron resource, namely hematite, was mixed with the coal char and the gasification pattern was then clarified. As the hematite was added into the coal char, different isothermal reaction phenomenon occurred compared to those with steel slags, as detailed in Fig. 5e–g. The sample mass started to decrease at relatively low temperatures (~850 °C) under Ar gas, which indicated that the Fe_2_O_3_ in hematite was reduced by the coal char, accounting for one of the traditional direct iron-making approaches^36^,^37^. In other words, from the viewpoint of Fe_x_O_y_ reduction by coal char, the hematite presented a higher activity than steel slags.

Similarly, to further clarify the effect of hematite on coal char gasification, a non-isothermal experiment was performed under pure CO_2_, as displayed in Fig. 5h. It can be clearly seen that, the coal char gasification was enhanced by the hematite due to the remarkably shortened reaction time, indicating a catalytic effect of hematite. On the other hand, there was no process of Fe_2_O_3_ reduction since the processing atmosphere was controlled as pure CO_2_. The aforementioned results provided two extreme situations, i.e., one is that the Ar gas was used when Fe_2_O_3_ was directly reduced into Fe by the char and the other is that the pure CO_2_ was employed when the valence state of the Fe_2_O_3_ did not change. Therefore it was reasonable that the Fe_2_O_3_ in the hematite could be first reduced into Fe_3_O_4_ as long as the reaction atmosphere was scientifically designed and controlled based on the Fe-C-O phase diagram in supplementary Fig. 4.

## Discussion

As aforementioned, through char gasification not only the thermal heat in the slags would be recovered but also the valence state of Fe would be changed, which could be further separated and extracted. After that, the residual solids mainly composed of CaO, SiO_2_, Al_2_O_3_, MgO and other minorities, similar to the chemical compositions of the Portland cement^15^,^16^,^39^, could be further used as raw materials in the cement industry after necessary modifications. Based on the foregoing clues, a big Fe-C-Ca cycle, comprising several individual loops, could be proposed with regard to several industrial systems.

**Figure 6:**
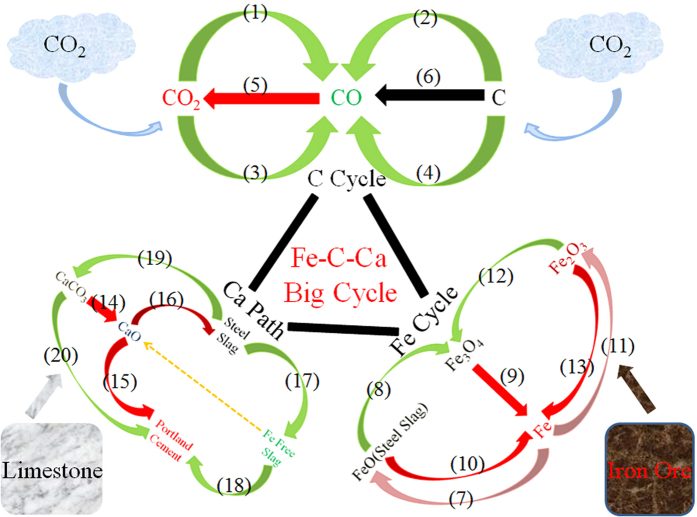
Fe-C-Ca big cycle proposed in this study. The processes and reactions involved in this big cycle include: I. **C-Cycle**. (1–2): 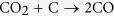; (3) 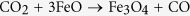; (4) 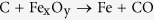; (5) 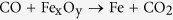; (6) 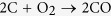. II. **Fe-Cycle**. (7) Steel Making: 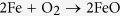; (8) 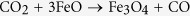; (9) 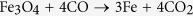; (10) 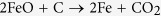; (11) Iron Rust: 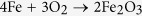; (12) 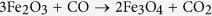; (13) 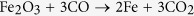. III. **Ca-Path**. (14) 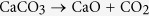; (15) Cement Production; (16) Steel Making Process; (17) Fe Extraction Process; (18) Cement Production; (19) CO_2_ Mineralization: 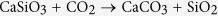; (20) Cement Production.

First, as one of the main objects was to utilize the iron resource herein, thus the initial loop making up the big cycle was Fe-cycle, as conceptually sketched in Fig. 6. It should be pointed out that both the hematite and the steel slags used in this study were typical iron resources and there could be two approaches to extract the iron in these minerals. The first approach was focused on Fe_3_O_4_ productions. The FeO in steel slags, produced by primary (from ore) or secondary (from scrap by iron rust) steel production, could be oxidized into Fe_3_O_4_ through exactly controlling the atmospheres, as exemplified by the coal char/steel slags gasification process in this study. The Fe_3_O_4_ formed could be then extracted via magnetic separation and further reduced by C or CO to produce pig iron. On the other hand, the FeO in steel slags and the Fe_2_O_3_ in hematite could be directly reduced by the carbon in the char and the pig iron was then produced, as exemplified by the coal char/hematite and the biomass char/steel slag reactions in this study. This method was relatively simple in technology, but large energy would be consumed due to the low content of Fe_x_O_y_ in these minerals^36^,^40^. These two ways, after necessary optimizations, provided the main strategies for utilizing the low-grade iron-bearing minerals in the future, which made up the key idea of the Fe-cycle.

Second, as one of the most important part of the Fe-C reaction, the C-cycle occupied a significant position, especially in the context of global warming mitigation, as schematized in Fig. 6. This small cycle was composed of several continuous steps. The carbon and CO_2_ could be first transformed into CO through char/CO_2_ gasification using the waste heat from steel slags and meanwhile, part of CO_2_ could be also reduced into CO by the FeO in steel slags, as demonstrated in this study. On the other hand, the CO could be oxidized into CO_2_ by the reaction of Fe_x_O_y_ reduction, which in fact, accounted for the typical iron-making process^36^,^40^.

Third, another important process in the big Fe-C-Ca-cycle was the Ca-path, as depicted in Fig. 6, since it referred to the CaCO_3_ calcination and thus the CO_2_ emission reduction. Firstly, as one of the raw materials for the chemical modification of steel slags, CaCO_3_ was calcined into CaO and the steel slags were generated with a high basicity (mass ratio of CaO to SiO_2_) of 2.0. Meanwhile, conventionally the CaO in the cement industry was generated by limestone calcination, contributing to an important source of CO_2_ emission in addition to fossil fuel combustion^41^,^42^. With regard to the chemical compositions of CaO, SiO_2_ and Al_2_O_3_, the steel slags in addition to blast furnace slags could be used in the cement industry after some pre-preparations such as iron extraction. Because of the high capacity of the cement industry and steel slags production, there is a great potential of energy saving and emission reduction. On the other hand, the present study was mainly focused on the direct utilization of steel slags as raw materials and there was another route for using the steel slags for emission reduction following the idea of carbon capture and storage (CCS), i.e., the steel slags were first modified for CO_2_ mineralization and then utilized as raw materials in the cement industry^43^,^44^. This strategy, in fact, provided another flow of Ca element in the big cycle while it did not affect the theoretical potential of emission reduction.

Considering these specific small loops, a big Fe-C-Ca cycle could be proposed herein, as schematized in Fig. 6 and based on this idea, the theoretical potential of energy saving, emission reduction and Fe production in modern carbon-intensive industries could be estimated. The energy saving following this system could be divided into three parts. The first part was the heat recovery from high temperature steel slags through char gasification. Considering the reaction activity, the gasification temperature was assumed to be higher than 1000 °C; in this case, the sensible heat of the slags was recovered in the temperature range of 1000–1550 °C. The output of steel slags in China was 123 Mt in 2014, and with the heat capacity of ~1.15 kJ/mol/K^9^,^14^, thus the heat recovery in this part was calculated as ~7.81 * 10^16^ J. The second part was the heat release during the FeO oxidization (Eq. (2)). It was estimated that the Fe_x_O_y_ content was 25%, of which around 65% was in the form of FeO^9^,^10^,^15^,^16^; accordingly the FeO production in steel slags was around 20 Mt and thus the heat release could be estimated as 1.55 * 10^15^ J. Additionally, in this stage ~4.09 * 10^9^ kg CO_2_ could be transformed into 2.60 * 10^9^ kg CO. These two parts of heat were defined as energy extraction, which would be further used as the heat source for char gasification. Using this heat, 2.10 * 10^10^ kg CO_2_ could be transformed into 2.68 * 10^10^ kg CO by the Boudouard reaction. For the third part, as the CaO in steel slags was reused in the cement industry, this would lead to a remarkable decrease of the CaCO_3_ calcination (Eq. (7)). CaO in steel slags was ~49 Mt and thus the energy saving and CO_2_ emission reduction achieved in the third part were estimated to be as much as 1.45 * 10^17^ J and 3.88 * 10^10^ kg, respectively.


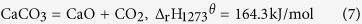


According to the foregoing calculations, the theoretical potentials for energy saving and emission reduction were equivalent to ~7.66 Mt standard coal and ~63.9 Mt CO_2_. Meanwhile, ~2.94 * 10^10^ kg CO was produced, which could be utilized into the ironmaking process through two ways, i.e., the reduction of the Fe_3_O_4_ recovered from the steel slags (Eq. (8)) and the reduction of Fe_2_O_3_ in hematite (Eq. (9)). The Fe_3_O_4_ recovered would be ~21.5 Mt, which could be reduced into ~15.6 Mt pig iron and the remaining CO after Fe_3_O_4_ reduction, could convert the Fe_2_O_3_ in hematite into ~25.2 Mt pig iron. Altogether the theoretical pig iron production achieved could be up to ~40.8 Mt in this big system.


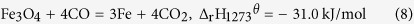



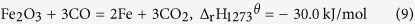


Given the foregoing analysis, the big Fe-C-Ca cycle provided a great potential of energy saving, CO_2_ emission reduction and iron recovery for modern industry. However, proposing a big system only remains a first step and this big vision to be realized, of course, requires a lot of technological innovations, to bring sustainability to the world surrounding us.

## Conclusions

In summary, to combat the issues of global warming and resource shortage, a big Fe-C-Ca cycle was proposed in this study for the first time. The disposal of a man-made iron resource, high temperature steel slags, using char/CO_2_ gasification was first investigated and the steel slags were found to show a great catalytic effect on char gasification. Meanwhile the FeO in the steel slags was oxidized into Fe_3_O_4_, which provided an important clue for iron resource recovery. Following this big cycle, it is expectative to realize the energy saving, emission reduction and resource recovery of 7.66 Mt of standard coal, 63.9 Mt of CO_2_ and 25.2 Mt of pig iron, respectively.

## Methods and Materials

### Sample preparation

To clarify the char gasification using the waste heat from steel slags, two typical kinds of char were first utilized. These char types were prepared from a coal and a biomass (wheat straw) in Shanxi Province, China, the proximate analysis and ultimate analysis results of which are detailed in Table 2. These chars were prepared by pyrolysis of the raw coal and biomass samples under Ar atmosphere at 1100 °C for more than two hours to confirm the thorough pyrolysis. On the other hand, two kinds of iron resources were adopted herein, i.e., an industrial steel slag and a hematite collected from Shougang Company, Beijing, China, the chemical compositions of which, measured by the X-ray fluorescence (XRF) technique, are listed in Table 3. This research was first focused on the treatment of steel slags using coal char gasification and to expand the utilization scale, the hematite and biomass char were further used. Before gasification, the samples were first crushed and ground into small particles (<200 mesh), dried at 105 °C for 24 hours and subsequently, thoroughly mixed using a ball grinder for 8 hours.

In addition, these raw materials were characterized using XRD (D/MAXPC 2500, Rigaku)) technique, as detailed in supplementary Fig. 5. In this study, altogether 7 samples were prepared and employed using the foregoing raw materials, i.e., a raw coal char (S1), a mixture of coal char and steel slags with the mass ratio of 1:1 (S2), a raw biomass char (S3), a mixture of biomass char and steel slags with the mass ratio of 1:1 (S4), a mixture of coal char and hematite with the mass ratio of 1:1 (S5), a raw steel slag (S6) and a raw hematite (S7).

### Gasification apparatus and process

A series of isothermal experiments were first performed to clarify the char gasification reaction and a gasification system was deployed mainly composed of a TG analyzer (S60/58341, Setaram) and a syngas analyzer (Testo pro350, Testo), as detailed in supplementary Fig. 6. The temperatures of the sample and the reactor in the TG analyzer were measured using thermocouples of type S (Pt-Rh10/Pt) following the temperature profile set in advance.

For each gasification run, ~10 mg char was weighed and placed in corundum crucible, with the height of 5 mm and diameter of 6 mm; meanwhile given the activity of the char gasification, the experimental temperature was selected as 1000 °C, 1100 °C, 1200 °C and 1300 °C. Overall, the whole gasification path could be divided into two steps. The char sample was first heated from room temperature to the set temperature at a rate of 10 °C/min with an argon (Ar) flow of 100 ml/min. After reaching the experimental temperature, it was held for 10 min under the Ar atmosphere for the stabilization of gas flow and temperature and the Ar gas was then replaced by CO_2_ gas with a flow rate of 100 mL/min. The char/CO_2_ gasification then took place at the pre-set temperature, during which the mass variation of the sample was continuously measured. To further identify char gasification process, some necessary non-isothermal experiments were also carried out and the agent was then adjusted, i.e., only pure CO_2_ gas was provided during the whole temperature schedule at a constant heating rate of 10 °C/min. Additionally, to deal with the buoyancy effect, blank runs were performed using empty crucibles or samples without char as backgrounds.

### Material and process characterization

To identify the transient process of char/slag gasification, scale-up experiments were thus performed. A larger amount of mixture of char and steel slags of ~5.0 g was rapidly placed in a tube furnace reactor, which was primarily heated to 1100 °C under CO_2_ flow. Then the gasified samples held at 1100 °C for different time intervals (0 min, 10 min, 40 min, 80 min and 120 min) were quickly pulled out of the tube and quenched under Ar atmosphere; the transient reaction state was thus retained and recorded. The solids obtained this way were characterized by XRD technique, which was operated under a voltage of 40 kV and a current of 100 mA and in the 2θ range of 10–80° with an increment of 0.02° and a scan speed of 4°/min.

### Methodology of kinetic mechanism of the char gasification process

After obtaining the mass evolutions of the samples, the isothermal kinetic mechanism of char gasification could be further characterized following several steps. First, the char conversion degree *x* versus time *t*, defined as the ratio of the sample consumed at time *t* to the final sample consumption, could be deduced based on the TG curves. Second, numerous mechanism functions previously developed, including Avrami-Erofeev models, shrinking core models and diffusion models as detailed in Supplementary Table 1^26^,^27^,^28^, were tentatively applied to fit theses conversion degree data by means of Eq. (10).


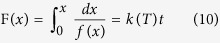


where *x*, *t*, *k*, *T*, *f*(*x*) and F(*x*) are the conversion degree, time, apparent gasification rate constant, absolute temperature, differential and integral mechanism functions, respectively.

Comparing the linear relationship of these plots, the reasonable mechanism models could be acquired and the apparent rate constants (*k*) for gasification could be further determined. Moreover, the elucidation of char gasification mechanism was performed based on three principles, i.e., the fundamental nature of each mechanism model, the knowledge demonstrated by previous studies and the mathematical optimum calculated herein. After attaining the rate constants, the apparent activation energy of gasification could be further calculated using Arrhenius equation (Eq. (11)).


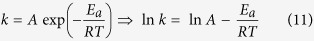


where *k*, *A*, *E*_*a*_, *R*, and *T* are the apparent rate constant, pre-exponential factor, apparent activation energy of gasification, gas constant, and absolute temperature (K), respectively.

## Additional Information

**How to cite this article**: Sun, Y. *et al.* A Fe-C-Ca big cycle in modern carbon-intensive industries: toward emission reduction and resource utilization. *Sci. Rep.* **6**, 22323; doi: 10.1038/srep22323 (2016).

## Supplementary Material

Supplementary Information

## Figures and Tables

**Table 1:** Kinetic models of coal char/CO_2_ gasification and the parameters deduced.

Kinetic Model	A3					D7				
Differential function Integral function	Avrami-Erofeev (m = 3) 3(1−x)[−ln(1−x)]^2/3^ [−ln(1−x)]^1/3^					3-D (Jander) 6(1−x)^2/3^[1−(1−x)^1/3^]^1/2^ [1−(1−x)^1/3^]^1/2^				
Sample	T/^o^C	R^2^-k calculation	k/min^−1^	R^2^-E_a_calculation	E_a_/kJ mol^−1^	T/^o^C	R^2^-k calculation	k/min^−1^	R^2^-E_a_ calculation	E_a_/kJ mol^−1^
S2	1000	0.9876	0.0343	0.9036	72.73	1000	0.9854	0.0221	0.9032	73.08
	1100	0.9980	0.0812			1100	0.9996	0.0526		
	1200	0.9974	0.1137			1200	0.9993	0.0737		
	1300	0.9960	0.1275			1300	0.9984	0.0827		
S1	1000	0.9656	0.0042	0.9609	185.05	1000	0.9566	0.0027	0.9616	185.21
	1100	0.9793	0.0212			1100	0.9728	0.0136		
	1200	0.9907	0.0756			1200	0.9862	0.0485		
	1300	0.9986	0.1065			1300	0.9994	0.0687		

**Table 2:** Proximate analysis and ultimate analysis of the chars employed.

Sample type	Proximate analysis (wt.%)				Ultimate analysis (wt.%)				
Composition	Moisture	Volatile	Ash	Fixed carbon	C	H	O*	N	HHV (MJ/kg)
Coal char	0.39	11.72	18.00	70.28	77.84	0.34	21.11	0.71	24.32
Biomass char	5.67	7.04	33.29	59.68	58.49	1.07	39.74	0.70	20.15

*Calculated by difference.

**Table 3:** Chemical compositions of the iron resources employed.

Composition (wt.%)	CaO	Fe_2_O_3_	SiO_2_	MgO	Al_2_O_3_	TiO_2_	P_2_O_5_	MnO	Fe^2+^/∑Fe
Steel slags	45.58	23.40	15.14	6.94	2.55	2.04	1.85	1.59	0.64
Composition (wt.%)	Fe_2_O_3_	SiO_2_	Al_2_O_3_	MgO	K_2_O	CaO	P_2_O_5_	MnO	TiO_2_
Hematite	51.43	18.40	7.91	2.68	1.73	1.10	0.38	0.33	0.23
