# Supplementary material for: A Fe-C-Ca big cycle in modern carbon-intensive industries: toward emission reduction and resource utilization
Source: Sci Rep. 2016 Feb 29;6:22323. doi: 10.1038/srep22323 (PMC4770321; doi:10.1038/srep22323)
Supplement: Supplementary Information [file srep22323-s1.pdf]

A Fe-C-Ca big cycle in modern carbon-intensive industries: toward emission reduction and resource utilization

Supplementary information

Yongqi Sun[1], Seetharaman Sridhar[2], Seshadri Seetharaman[3], Hao Wang[1], Lili Liu[1], Xidong Wang[1] and Zuotai Zhang[1,4*]

1 Department of Energy and Resources Engineering, College of Engineering, Peking University, Beijing 100871, P.R. China；

2 WMG, International Digital Laboratory, University of Warwick, Coventry CV4 7AL, UK；

3 Department of Materials Science and Engineering, Royal Institute of Technology, Stockholm, Vallslingan 14, SE-187 52 Täby, Sweden

4 School of Environmental Science and Engineering, South University of Science and Technology of China, Shenzhen, P.R.China

Supplementary Table 1. Various Gas-Solid kinetic mechanism functions, which could be divided into three types, namely Avrami-Erofeev models, shrinking core models, and diffusion models.

No	Kinetic mechanism	Differential function: f(x)	Integral function: F(x)
A_m	Avrami-Erofeev	$m(1-x)[-\ln(1-x)]^{m-1/m}$	$[-\ln(1-x)]^{1/m}$
A_1	m=1	1-x	$-\ln(1-x)$
A_2	m=2	$2(1-x)[-\ln(1-x)]^{1/2}$	$[-\ln(1-x)]^{1/2}$
A_3	m=3	$3(1-x)[-\ln(1-x)]^{2/3}$	$[-\ln(1-x)]^{1/3}$
A_4	m=4	$4(1-x)[-\ln(1-x)]^{3/4}$	$[-\ln(1-x)]^{1/3}$
S_m	Shrinking core	$m(1-x)^{m-1/m}$	$1-(1-x)^{1/m}$
$S_{1/2}$	m=1/2	$(1/2)(1-x)^{-1}$	$1-(1-x)^2$
$S_{1/3}$	m=1/3	$(1/3)(1-x)^{-2}$	$1-(1-x)^3$
$S_{1/4}$	m=1/4	$(1/4)(1-x)^{-3}$	$1-(1-x)^4$
S_2	m=2	$2(1-x)^{1/2}$	$1-(1-x)^{1/2}$
S_3	m=3	$3(1-x)^{2/3}$	$1-(1-x)^{1/3}$
D_m	Diffusion model		
D_1	one-dimensional	$1/2x^{-1}$	x^2
D_2	two-dimensional	$[-\ln(1-x)]^{-1}$	$x+(1-x)\ln(1-x)$
D_3	three-dimensional	$(3/2)(1-x)^{2/3}[1-(1-x)^{1/3}]^{-1}$	$[1-(1-x)^{1/3}]^2$
D_4	three-dimensional	$(3/2)[(1-x)^{-1/3}-1]^{-1}$	$1-2/3x-(1-x)^{2/3}$
D_5	3-D (anti-Jander)	$(3/2)(1+x)^{2/3}[(1+x)^{1/3}-1]^{-1}$	$[(1+x)^{1/3}-1]^2$
D_6	3-D (ZLT)	$(3/2)(1-x)^{4/3}[(1-x)^{-1/3}-1]^{-1}$	$[(1-x)^{-1/3}-1]^2$
D_7	3-D (Jander)	$6(1-x)^{2/3}[1-(1-x)^{1/3}]^{1/2}$	$[1-(1-x)^{1/3}]^{1/2}$
D_8	2-D (Jander)	$(1-x)^{1/2}[1-(1-x)^{1/2}]^2$	$[1-(1-x)^{1/2}]^2$
C_n	Chemical reaction	$(1-x)^n$	$(1-(1-x)^{1-n})/(1-n)$
C_1	n=2	$(1-x)^2$	$(1-x)^{-1}-1$
C_2	n=3/2	$2(1-x)^{(3/2)}$	$(1-x)^{-1/2}-1$

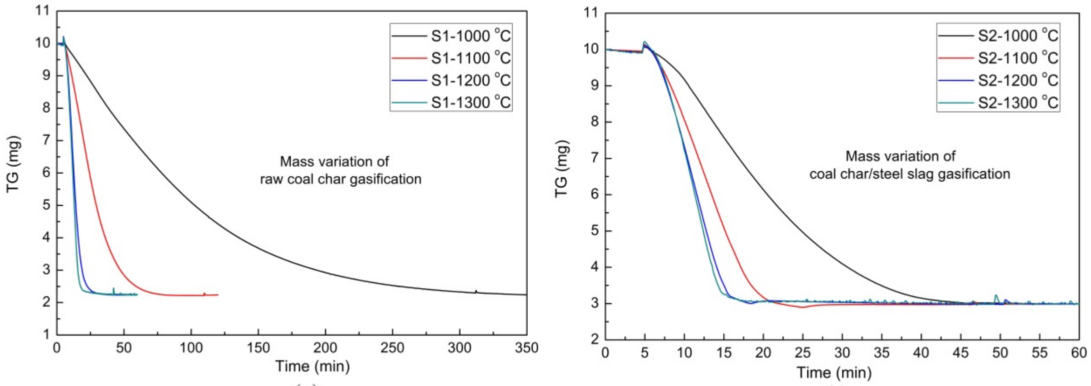

(a) (b)

Supplementary Figure 1. Mass evolutions of the samples during isothermal coal char gasification reactions with varying gasifying temperatures. (a) Coal char without steel slags (**S1**) and (b) coal char with steel slags (**S2**).

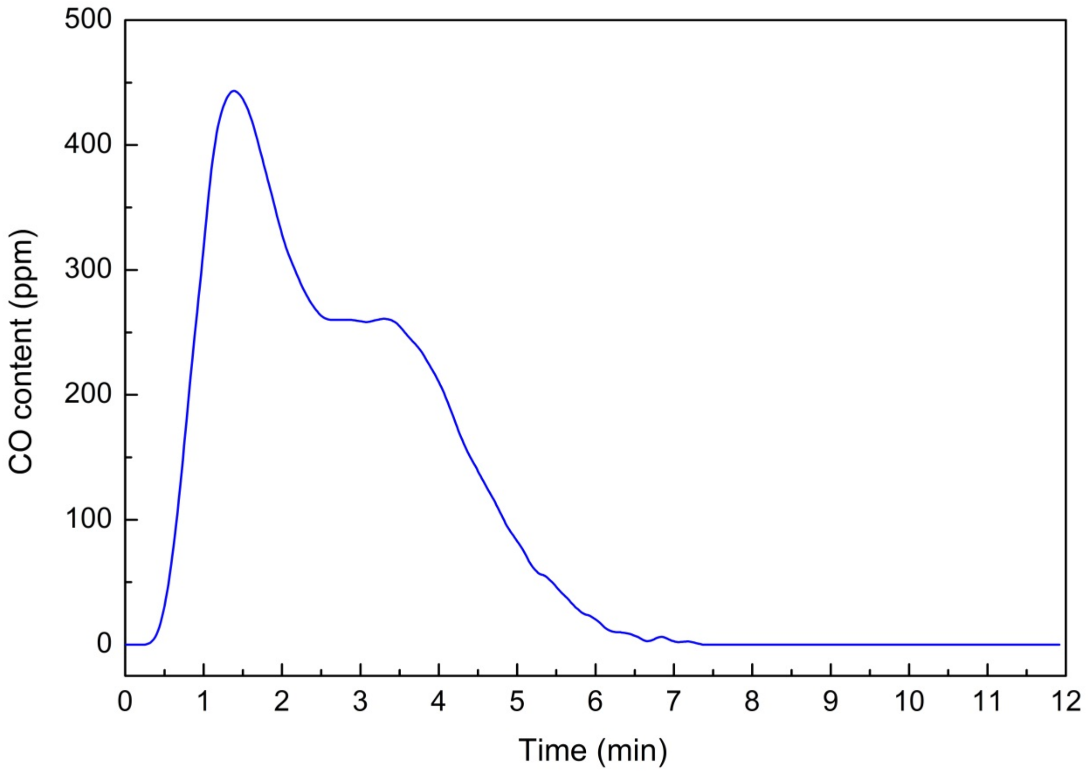

Supplementary Figure 2. CO content versus time when the steel slags were heated at 1100 ^{o}C in the agent of CO_2.

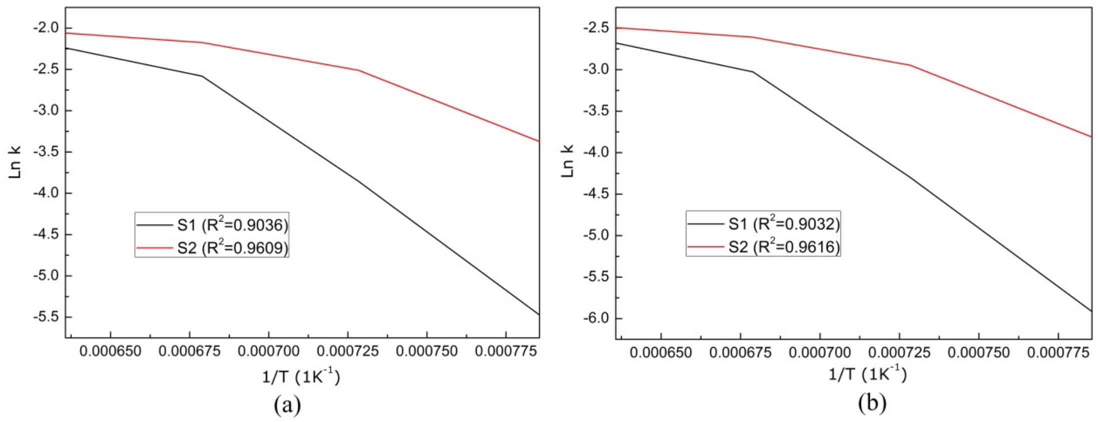

Supplementary Figure 3. Calculations of the activation energy for coal char gasification reactions. (a) A3 model and (b) D7 model. Sample **S1**: raw coal char; Sample **S2**: coal char mixed with steel slags.

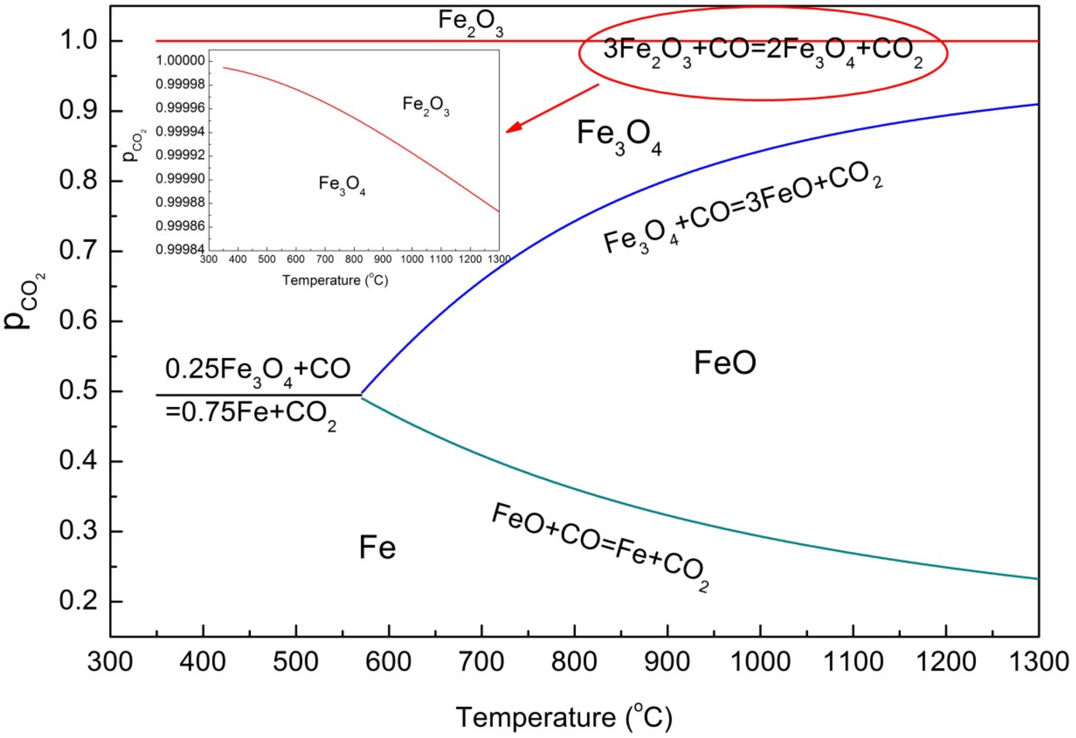

Supplementary Figure 4. Fe-C-O phase diagram. This clarifies the main formation of iron oxide phases with varying temperature and CO_2 pressures in theory.

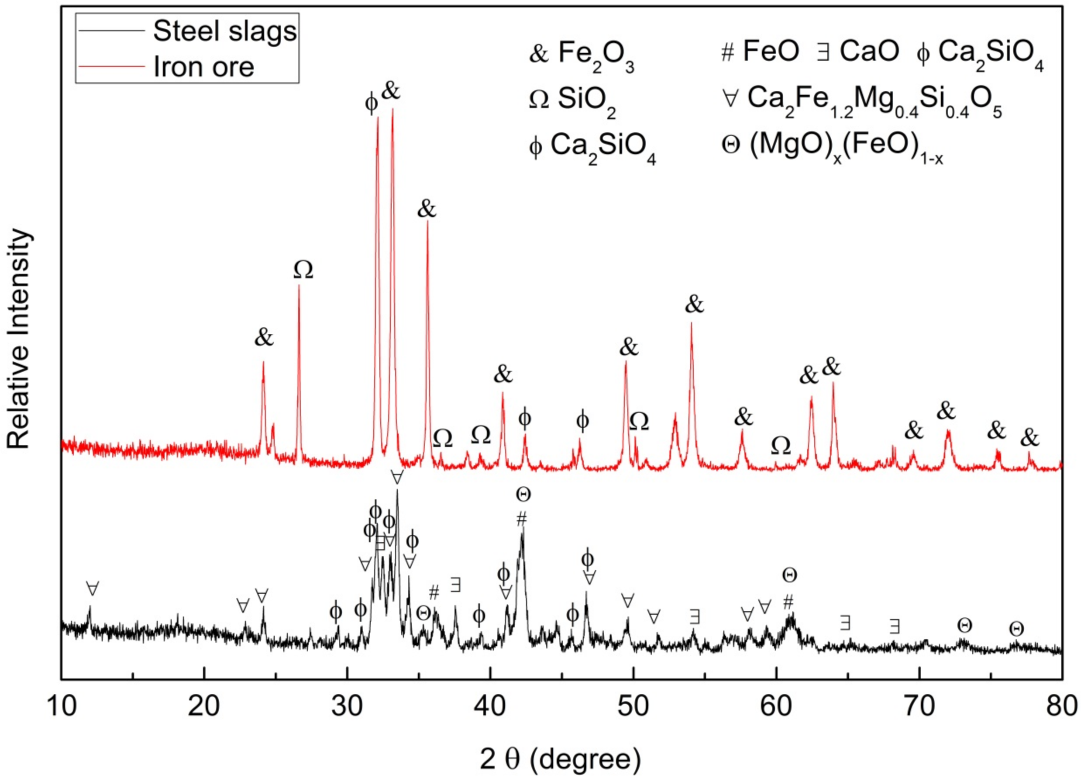

Supplementary Figure 5. XRD characterizations of the steel slags and hematite used in this study.

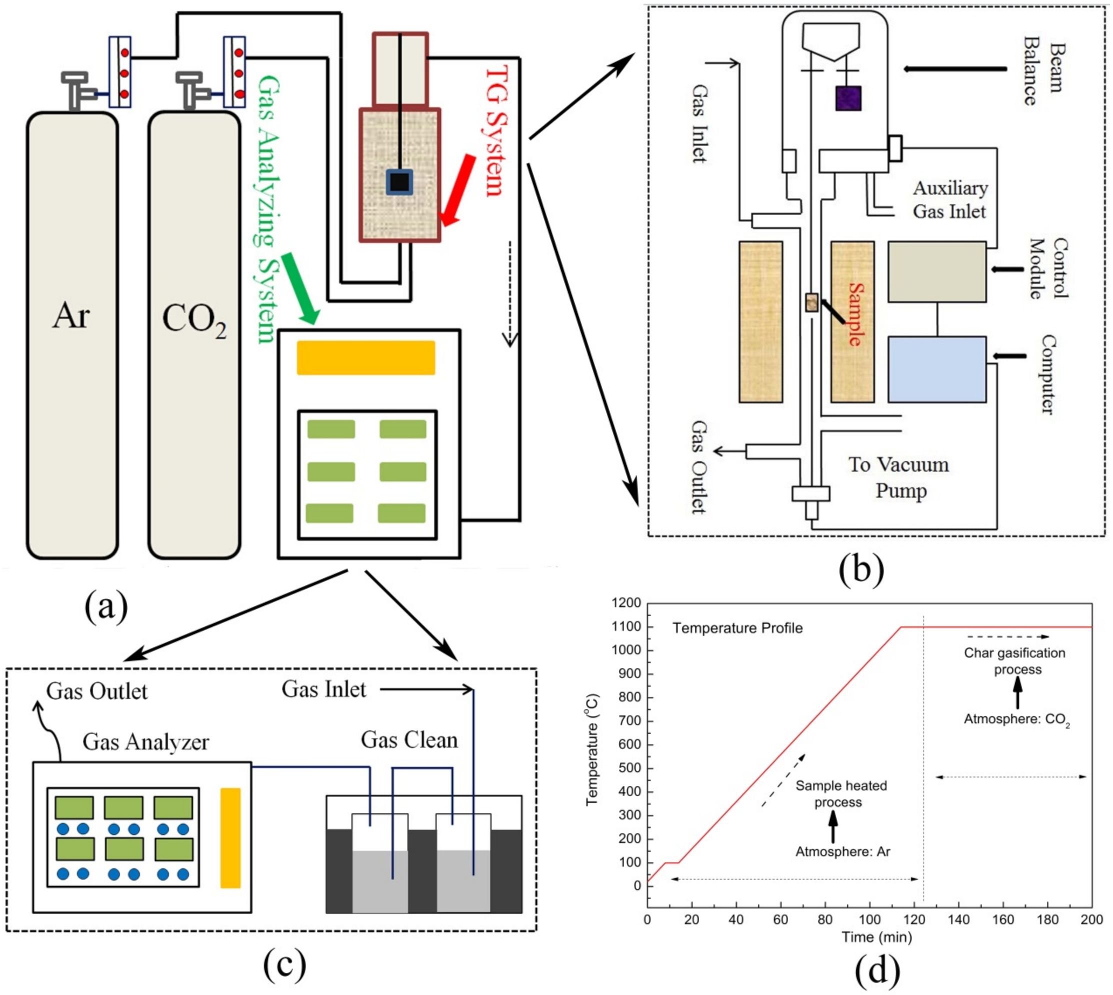

Supplementary Figure 6. Schematic of char gasification system, which could be divided into two parts: a TG analyzers part and a gas cleaner and analyzer part. (a) total system, (b) char gasification part, (c) gas analysis part and (d) temperature files of the gasification process.
